# Individual functional parcellation revealed compensation of dynamic limbic network organization in healthy ageing

**DOI:** 10.1002/hbm.26096

**Published:** 2022-10-10

**Authors:** Tiantian Liu, Zhongyan Shi, Jian Zhang, Kexin Wang, Yuanhao Li, Guangying Pei, Li Wang, Jinglong Wu, Tianyi Yan

**Affiliations:** ^1^ School of Life Science Beijing Institute of Technology Beijing China; ^2^ Intelligent Robotics Institute, School of Mechatronical Engineering Beijing Institute of Technology Beijing China; ^3^ School of Medical Technology Beijing Institute of Technology Beijing China

**Keywords:** brain states, clustering, dynamic functional connectivity, individual sliding window, resting‐state fMRI

## Abstract

Using group‐level functional parcellations and constant‐length sliding window analysis, dynamic functional connectivity studies have revealed network‐specific impairment and compensation in healthy ageing. However, functional parcellation and dynamic time windows vary across individuals; individual‐level ageing‐related brain dynamics are uncertain. Here, we performed individual parcellation and individual‐length sliding window clustering to characterize ageing‐related dynamic network changes. Healthy participants (*n* = 637, 18–88 years) from the Cambridge Centre for Ageing and Neuroscience dataset were included. An individual seven‐network parcellation, varied from group‐level parcellation, was mapped for each participant. For each network, strong and weak cognitive brain states were revealed by individual‐length sliding window clustering and canonical correlation analysis. The results showed negative linear correlations between age and change ratios of sizes in the default mode, frontoparietal, and salience networks and a positive linear correlation between age and change ratios of size in the limbic network (LN). With increasing age, the occurrence and dwell time of strong states showed inverted U‐shaped patterns or a linear decreasing pattern in most networks but showed a linear increasing pattern in the LN. Overall, this study reveals a compensative increase in emotional networks (i.e., the LN) and a decline in cognitive and primary sensory networks in healthy ageing. These findings may provide insights into network‐specific and individual‐level targeting during neuromodulation in ageing and ageing‐related diseases.

## Graphical abstract

The present study performed individual functional parcellation and individual‐length sliding window clustering on resting‐state functional magnetic resonance imaging data (637 participants in total, 18–88 years) and characterized ageing‐related dynamic network changes. The results showed atrophy and U‐shaped patterns of most cognitive functional networks but expansion and inverted U‐shaped patterns of emotion‐related limbic network in healthy ageing. These findings provide new insight into the understanding of cognitive ageing.

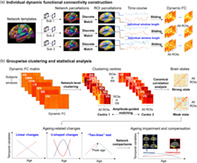

## 1 INTRODUCTION

Healthy ageing is typically characterized by subtle declines and slowing in general cognitive abilities, which may further progress into pathological impairment and even dementia. The development of noninvasive neuroimaging methods, such as various magnetic resonance imaging (MRI) sequences, has made it possible to detect structural and functional brain markers in ageing, such as regional cortical thickness (Frangou et al., 2022), white matter integrity (Miller et al., 2016), and functional network organization (Power et al., 2011). Structural brain changes have been extensively studied in the context of healthy ageing and related neurodegenerative disorders (Yan et al., 2018) but are assumed to occur later than functional changes (Jack et al., 2010; Zonneveld et al., 2019). Functional studies, especially studies on functional network organization, could help motivate investigations of or interventions for ageing‐related neurodegenerative disorders, such as Alzheimer's disease (AD) (B. Wang et al., 2017; Xu et al., 2021). For example, functional networks can serve as individual intervention targets in healthy older adults and AD patients when using noninvasive neuromodulation to enhance cognitive functions (Nilakantan et al., 2019).

Resting‐state functional MRI (rs‐fMRI) measures intrinsic low‐frequency (<0.1 Hz) blood oxygenation level‐dependent (BOLD) signal activities (Cordes et al., 2001) and has revealed large‐scale functional networks, including those linked to high‐order cognitive and emotional functions (default mode [DMN], attentional [AN], salience [SN], frontoparietal [FPN], and limbic [LN] networks) and those supporting primary sensory functions (visual [VN] and motor‐sensory [MN] networks) (Yeo et al., 2011). These networks are organized to support different brain cognitive functions by segregating and integrating within and between networks (Yan et al., 2022; H. Y. Zhang et al., 2021) and have revealed neural dedifferentiation in healthy ageing, which indicates decreased independence, decreased segregation of functional networks and inability to specify relevant neural circuits to mediate specialized functional processes (Chan et al., 2014; King et al., 2018). Static functional connectivity (FC) analysis measures statistical temporal correlations of mean BOLD time series signals between distinct brain regions across the entire scanning time and has shown that the ageing brain undergoes complex functional reorganization and compensation (H. Zhang et al., 2017; Zonneveld et al., 2019). Ageing‐related reorganization is characterized by weaker within‐network connectivity and controversial between‐network findings, that is, greater between‐network connectivity (Geerligs et al., 2015; Spreng & Turner, 2019; Zonneveld et al., 2019) or weaker between‐network connectivity (Varangis et al., 2019; H. Y. Zhang et al., 2021).

Using a “dynamic” analysis, in which time‐varying, dynamic FC is measured within a series of overlapping “sliding windows” in the BOLD timeseries data (Preti et al., 2017), studies have found that older adults spend more time in a baseline, weak connectivity state (Tian et al., 2018), indicating loss of dynamics and the inability to adapt to environmental variations (Garrett et al., 2021). In addition, older adults may spend less time in a series of different states, as revealed by the following discrepant findings. The brain state may be characterized by antagonistic activity of the DMN and AN (K. Y. Chen et al., 2022), by high connectivity within the MN and the cognitive control network (Tian et al., 2018), or by many positive connections among subnetworks (Xia et al., 2019). Overall, static between‐network connectivity and dynamic functional network state findings are controversial, and we suspect that these discrepancies are due to the use of different analytic methods, especially node or region of interest (ROI) spatial definitions, when constructing individual functional networks.

Like many other complex networks, nodes and edges are two basic elements in brain functional networks. Generally, nodes are typically defined by ROIs in a predefined, group‐average and nonoverlapping resting‐state network (RSN) parcellation. Edges, also named FCs, are typically defined by Pearson or partial correlation coefficients of averaged BOLD time series in ROIs. However, of particular importance, different RSN parcellations may assign the same voxel or vertex to different networks, especially voxels (or vertices) in subcortical networks (Doucet et al., 2019). In addition, most group‐level parcellations are derived from young adult (age <40 years) data (Gordon et al., 2016; Power et al., 2011; Yeo et al., 2011) but are not suitable for participants across the lifespan. Inspired by this question, one study mapped individual functional parcellation and revealed location reconfiguration of functional regions in ageing adults (Geerligs et al., 2017), indicating inaccurate calculation of node signals and FC using young adult brain parcellations. Recent studies have focused on age‐appropriate brain parcellation, including an age‐appropriate functional parcellation derived from older adults, ages ranging from 55 to 95 years (Doucet et al., 2021), and five cohort‐specific parcellations (age range: 20–34, 35–49, 50–64, 65–79, and 80–93 years). However, no matter what the group‐level parcellation is, averaging individual BOLD signal data based on group‐level network parcellation (Braga & Buckner, 2017) may underestimate certain participant‐specific properties and details of functional network architecture, such as cross‐individual variations in the shape, size, and position of functional networks (Bijsterbosch et al., 2018). Therefore, it is necessary to map individual‐level parcellation and further analyse ageing‐related changes based on it.

Recent methodological breakthroughs, in which individual‐level parcellation has been mapped using iterative clustering analysis on group‐level parcellations (M. L. Li et al., 2019; D. H. Wang et al., 2015), offer the opportunity to characterize dynamic functional network organization changes with age at the individual level. In addition, researchers have noticed the drawback and limitation of fixed‐length sliding window analysis and proposed data‐driven segmentation of sliding windows (Choe et al., 2017), such as the hidden Markov model (G. M. Zhang et al., 2020), the dynamic conditional correlation model (Lindquist et al., 2014), and activation‐informed temporal segmentation (Duda et al., 2021). Considering differences in interindividual parcellation, internetwork organization and interindividual sliding window activities with increasing age, we used individual participant parcellation and individual network dynamic analysis on individual sliding window lengths to advance the understanding of dynamic network organization. In addition, we correlated recurring dynamic brain states with multiple cognitive behavioural measures to assign different states to corresponding cognitive levels. According to the compensation hypothesis (Cabeza et al., 2018; Reuter‐Lorenz & Park, 2014), we expected to find inverted “U‐shaped” correlations (impairment) between high cognitive level brain states and age in most networks and “U‐shaped” correlations (compensation) in specific networks.

## 2 MATERIALS AND METHODS

### 2.1 Dataset

Neuroimaging data from 639 cognitively healthy participants were studied as part of the Cambridge Center for Ageing and Neuroscience (Cam‐CAN) project (Stage 2 cohort, available at http://www.mrc-cbu.cam.ac.uk/datasets/camcan/) (Shafto et al., 2014; Taylor et al., 2017). The included participants were not diagnosed with diseases that would impact brain functions, such as dementia, AD, Parkinson's disease, multiple sclerosis, stroke, or epilepsy (more exclusion criteria are shown in Shafto et al. (2014)). Two participants were excluded from the analyses due to excessive head motion (details below), resulting in 637 participants (18–88 years old, mean ± SD age = 54.25 ± 18.45 years; 311 males and 326 females) included in the final sample. All participants gave written informed consent, and the Cambridgeshire 2 Research Ethics Committee approved the study. Participants' exclusion criteria of Cam‐CAN and more details are shown in a previous publication (Shafto et al., 2014).

For each participant, T1 structural and resting‐state BOLD fMRI data were acquired on a 3 T Siemens TIM Trio System using a 32‐channel head coil. The high‐resolution T1‐weighted structural images were acquired using a magnetization prepared rapid gradient echo sequence, with repetition time (TR) = 2250 ms, echo time (TE) = 2.99 ms, inversion time (TI) = 900 ms, FA = 9°, field of view (FOV) = 256 × 240 × 192 mm^3^, and voxel size = 1 × 1 × 1 mm^3^. Rs‐fMRI images were acquired using an echo‐planar imaging sequence, with TR = 1970 ms, TE = 30 ms, flip angle = 78°, FOV = 192 × 192 mm^2^, voxel size = 3 × 3 × 4.44 mm^3^, slice thickness = 3.7 mm, and slice number = 32. During individual rs‐fMRI scanning (8 min and 40 s, 261 volumes), participants were instructed to rest with their eyes closed.

In addition, this study included 13 cognitive variables derived from 8 outside‐MRI cognitive tasks, including the fluid intelligence task, the hotel task, the picture–picture priming task, the proverb comprehension task, the visual short‐term memory task, the choice motor coefficient of variation task, the face recognition task, and the emotion expression recognition task. These cognitive tasks are used to evaluate five cognitive domains, including executive functions, language functions, memory function, motor function, and emotional processing. A brief description of each variable is summarized in Table 1, and full descriptions are given in a previous publication (Shafto et al., 2014).

**TABLE 1:** Description of cognitive tasks (table adapted from Tibon et al. (2021) and Taylor et al. (2017))

Cognitive domain	Cognitive task	Variables	Descriptive statistics for *N* = 637 (Mean, *SD*)
Executive functions	Fluid intelligence	Total score on the Cattell task	Mean = 31.92, *SD* = 6.71
	Hotel task	Deviation from optimum time allocation on the hotel task	Mean = 305.31, *SD* = 173.29
Language functions	Picture–picture priming	Number of trials with RT > 200 ms and correct response (not hesitation)	Mean = 0.68, *SD* = 0.21
	Proverb comprehension	Sum of three scores (0: incorrect, 1: concrete, 2: abstract)	Mean = 4.52, *SD* = 1.62
Memory function	Visual short‐term memory	Number of reportable items with four coloured discs	Mean = 2.51, *SD* = 0.81
Motor function	Choice motor coefficient of variation	Coefficient of variation for all trials, response times were inverted (1/RT) before the response time measures were computed, response times 3 *SD* above or below the mean were removed before computing the response time measures	Mean = 0.19, *SD* = 0.06
Emotional processing	Face recognition	Total score on the Benton faces task	Mean = 22.91, *SD* = 2.36
	Emotion expression recognition	Accuracy of anger expression	Mean = 80.64, *SD* = 23.94
		Accuracy of disgust expression	Mean = 84.44, *SD* = 21.08
		Accuracy of fear expression	Mean = 73.86, *SD* = 23.55
		Accuracy of happy expression	Mean = 96.34, *SD* = 11.56
		Accuracy of sad expression	Mean = 90.93, *SD* = 17.28
		Accuracy of surprise expression	Mean = 88.20, *SD* = 14.87

Abbreviation: *SD*, standard deviation.

### 2.2 Data preprocessing

Rs‐fMRI data were preprocessed using FSL (https://fsl.fmrib.ox.ac.uk/fsl/fslwiki/) with the following steps: discarding the first 10 volumes, correcting head motion by MCFLIRT, slice timing correction, extracting nonbrain tissues with BET, spatial smoothing with full width at half maximum = 6 mm, normalizing intensity, high‐pass temporal filtering (cut‐off frequency = 0.01 Hz) and registering the rs‐fMRI to high‐resolution T1‐weighted structural images. To exclude the head motion effects, head movements exceeding 2 mm or 2° in any direction were discarded (*n* = 2). The final sample included 637 participants. In addition, the covariate head motion was calculated as the average of the root mean squared realign parameters at all 251 time points using MCFLIRT.

T1‐weighted structural images were processed using the FreeSurfer version 6.0.0 software package. Total cortical grey matter volume was calculated as a covariate in statistical analyses (details below). The structural and functional images were aligned using boundary‐based registration. Rs‐fMRI data were aligned to a spherical coordinate system by sampling from the cortical ribbon in a single interpolation. The rs‐fMRI data of each individual were first registered to the FreeSurfer surface template, which consisted of 40,962 vertices in each hemisphere. The smoothed data were then downsampled to a mesh of 2562 vertices in each hemisphere using the mri_surf2surf function in the FreeSurfer software package.

### 2.3 Individual parcellation and functional network size calculation

**FIGURE 1:**
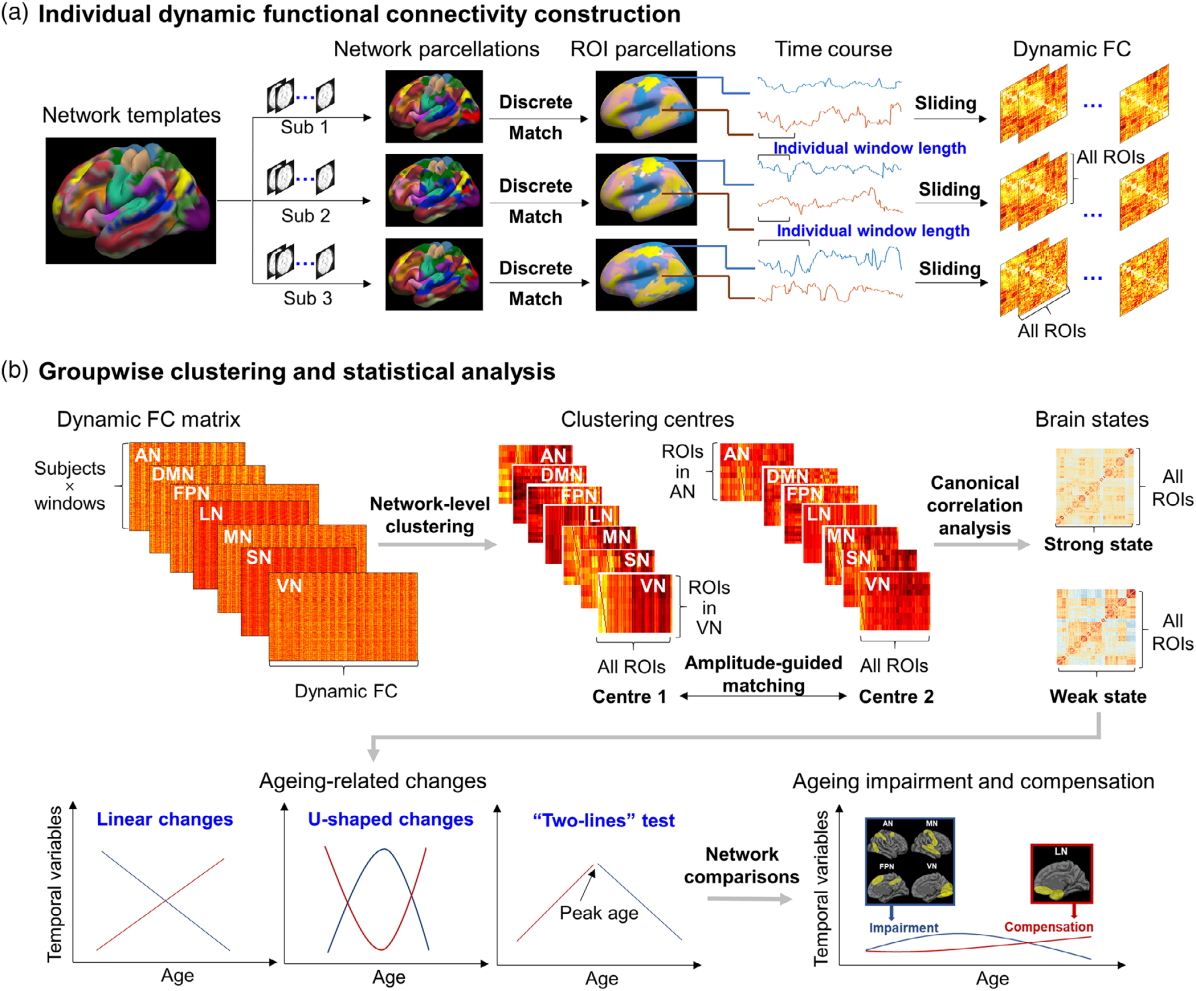
Workflow of analysis. (a) Individual dynamic functional connectivity construction. Group‐level network templates including seven networks were extracted from a previous publication (Yeo et al., 2011). Individual preprocessed functional magnetic resonance imaging (fMRI) time courses were iteratively clustered based on the group network template to generate individual network parcellation. Then, the network parcellation was separated into the region of interest (ROI) parcellation. ROI signals were extracted to calculate dynamic functional connectivity (FC) using individual sliding windows. (b) Groupwise clustering and statistical analysis. Dynamic FC within each network of all subjects was combined into a feature matrix. Clustering analysis was performed on these seven network feature matrices, resulting in clustering centres for each participant. Using canonical correlation analysis, clustering centres were grouped into the strong state and weak state. Finally, groupwise changes with age were calculated and summarized to determine ageing impairment and compensation. AN, attentional network; DMN, default mode network; FPN, frontoparietal network; LN, limbic network; MN, motor‐sensory network; SN, salience network; VN, visual network

Individual parcellation analysis was performed using the HFR_ai toolbox with the following steps (M. L. Li et al., 2019) (Figure 1). First, guided by the group‐level 18 functional network parcellation derived from 1000 healthy participants (M. L. Li et al., 2019; D. H. Wang et al., 2015; Yeo et al., 2011), an iterative parcellation algorithm was applied to individual surface‐projected rs‐fMRI data, and each vertex on the cortical surface was assigned to one of 18 networks, resulting in 18 individual network parcellations for every participant. Second, individual‐level cortical network parcellations were segmented into discrete patches using a clustering algorithm. Third, the patches were matched to 116 cortical ROIs extracted from 18 group‐level networks, resulting in individual ROI parcellations. It is important to note that if a patch did not overlap with any cortical ROI and was not near any ROI, the patch would be labelled “unrecognized.” Thus, the number of individual cortical ROIs was less than or equal to 116. Considering the intersection of individual ROI parcellation, only 27 homologous ROIs were retained for 100% of participants, and the number of ROIs was too small to analyse individual functional variations. Therefore, we kept ROIs that were defined for 90% of participants. Finally, 88 ROIs were kept for subsequent analysis.

To better summarize between‐participants functional variations in network size and compare them with previous findings, we further grouped the 18 networks into seven well‐studied functional networks, including the AN, DMN, FPN, MN, LN, SN, and VN. Given any of seven networks, the change ratio of the network size (*S*_*r*_) was quantified as follows:

$$S_{r}=\frac{\sum_{k=1}^{m}l_{k}-\sum_{j=1}^{m}l_{j}}{\sum_{j=1}^{m}l_{j}}$$

where *m* indicates the number of vertices in the given network, *l*_*k*_ indicates the assigned label of vertex *k* within individual‐level ROI parcellation, and *l*_*j*_ indicates the assigned label of vertex *j* within group‐level ROI parcellation. The assigned label was 1 or 0. Finally, the change ratio of the network size was calculated for each network for each participant.

### 2.4 Clustering‐based dynamic functional state construction

For each participant, 251 volumes (time points), with 88 ROIs in each volume, were included in the following analysis (Figure 1). At each time point, the average of the preprocessed BOLD signal across all vertices in a given ROI was extracted. Considering the different resting‐state FC patterns of different networks, we separately applied a sliding window and clustering analysis to each of the seven networks. Given one network and one participant, dynamic FC was calculated using a sliding window method, with varied window length (ranging from 18 TR to 28 TR) and fixed sliding step length (1 TR). The optimum window length (*L*) at time point *t* was defined as follows:

$$L\left(t\right)=\max_{a}\left(\sum_{\mathrm{roi}=1}^{n}x\left(t+a-1,\mathrm{roi}\right)\right)$$

where *x* indicates whether the given ROI signal at time point (*t* + *a* − 1) was local extrema. If the ROI signal was larger (or lower) than the signal at the former and the latter time points, the ROI signal at the current time point was a local extremum, and *x* was equal to 1; otherwise, *x* was equal to 0, *a* indicates a different window length (range from 18 to 28), and *n* indicates the number of ROIs within a given network. The number of ROIs (*n*) was equal to 10, 17, 23, 4, 14, 10, and 10 for the AN, DMN, FPN, MN, LN, SN, and VN, respectively. Finally, the optimum window length kept most local extrema (variations) within the given network.

For consistency among different participants, the first to 224th consecutive windows were used for the following analysis. In each time window, Pearson correlation coefficients were calculated for pairwise ROI signals to obtain FC measures. To improve the normality of the correlation coefficient matrix, we transformed the matrix into *z scores* using Fisher's *r*‐to‐*z* transformation. Finally, we obtained a 224 × 88 × 88 dynamic FC matrix for each participant.

The *k*‐means clustering algorithm was employed to classify the FC matrix into different groups based on similarities and to capture the reoccurring FC patterns across time and participants. A total of 142,688 (637 participants × 224 windows) samples were used as observations, and off‐diagonal elements in the FC matrix were used as features. In detail, 10 ROIs were included within the VN, so all FCs related to those 10 ROIs were extracted as the input of VN *k*‐means clustering. Therefore, seven rounds of clustering were performed on dynamic FC of the AN (10 × 87 features), DMN (17 × 87 features), FPN (23 × 87 features), LN (4 × 87 features), MN (14 × 87 features), SN (10 × 87 features), and VN (10 × 87 features). When performing clustering analysis, the Manhattan distance (*L*_1_ norm) was used to estimate the similarity among FC matrices because the Manhattan distance was more preferable than the Euclidean distance (*L*_2_ norm) for the case of high‐dimensional data (Aggarwal et al., 2001). To improve the stability of clustering, we iterated *k*‐means clustering 100 times. Since clustering analysis was performed on each network and clustering centres were matched for all networks, *k* was set to 2 to match the clustering results with low deviation. In addition, two clusters were widely reported in previous fMRI studies (Kim et al., 2017; H. Li et al., 2022; D. Zhang et al., 2022; Zheng et al., 2022). Finally, clustering algorithms labelled FC matrices into one of the two clusters, and the median FC in the same cluster was calculated as a cluster centroid. Here, the cluster centroids were defined as brain states.

### 2.5 Dynamic functional state analysis

To depict the characteristics of dynamic functional states, amplitude was defined as the average of absolute FC in each state. In addition, we calculated two clustering indexes to compare the temporal metrics from each participant's state vector, including (1) dwell time, which was calculated by the maximum consecutive windows assigned to the same state and represented how long the participant stayed in the state continuously; and (2) occurrence, which was calculated by the number of windows assigned to one state. For each participant, clustering of each network resulted in two amplitudes, two dwell times, and two occurrence values.

### 2.6 Statistical analysis

To exclude the influences of covariates, we first constructed a linear model, with each metric (including the change ratio of the network size, amplitude, dwell time, occurrence, and transition time) as the dependent variable and covariates (including head motion, sex, and cortical grey matter volume) as independent variables. The resulting residuals were used to construct linear and quadratic regression models of age with a significance level of *p* < .05. Then, the Akaike information criterion (AIC) was used to compare the goodness of fit of linear and quadratic regression models. When exhibiting quadratic relations (U‐shape or inverted U‐shape), according to a previous study (K. Y. Liu et al., 2019), a post hoc “two‐lines” test was performed to assess whether there was a point where the slope of function was changed. The point was then defined as peak age. In addition, differences among networks, including size, amplitude, dwell time, occurrence, and transition time, were tested using a generalized estimated equation (GEE), with head motion, sex, and cortical grey matter volume as covariates.

The Kolmogorov–Smirnov test was used to assess normality, and amplitude differences in the two clustering states were tested by the Wilcoxon rank sum test. Since multiple variates were included in amplitudes of each state (7 networks) and cognitive task (13 variables), correlations between amplitudes and cognitive tests were modelled using canonical correlation analysis (CCA) (Tibon et al., 2021), a powerful method to simultaneously examine linear relationships between multiple amplitudes and cognitive tasks derived from each participant. Then, the statistical comparison of correlation coefficients of CCA was performed using the “cocor” package (Diedenhofen & Musch, 2015).

The significance level was set to *p* < .05, and a false discovery rate correction was applied to correct multiple correlations. The above statistical analyses were performed using MATLAB R2014a, R 4.1.2 or SPSS 23. GEE was performed using SPSS 23, a U‐shaped relationship post hoc “two‐lines” test was performed using R 4.1.2, and other analyses were performed using MATLAB R2014a.

## 3 RESULTS

### 3.1 Individual parcellation and network size differences

**FIGURE 2:**
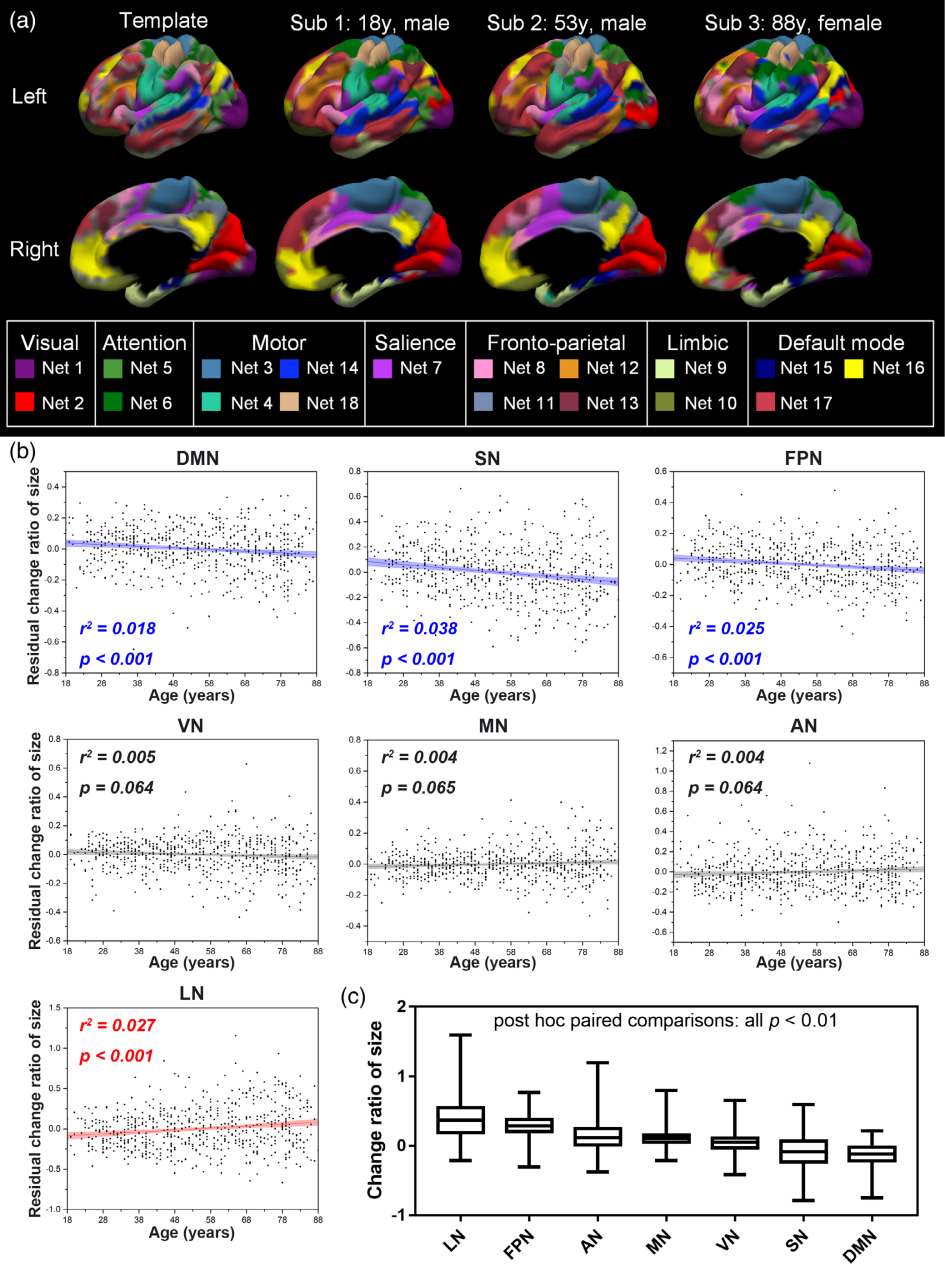
Individual network parcellations and ageing changes. (a) Group network parcellations and three participants' network parcellations (Sub 1, Sub 2, and Sub 3). Networks are shown in different colours. (b) Network size changes with age. Linear regression was performed on the size of seven networks and age, with sex, head motion, and cortical grey matter volume as covariates. Significant linear decrease (DMN, SN, FPN) and increase (LN) relationships with age are plotted in blue and red, respectively. The change ratio of size in VN, MN and AN showed no significant relationships and are plotted in black. False discovery rate (FDR) correction was applied for multiple comparisons. (c) The change ratio of network size is shown in box and whisker plots. Generalized estimated equation (GEE) analysis showed significant differences between each pair of networks (all *p* < .01). AN, attentional network; LN, limbic network; DMN, default mode network; FPN, frontoparietal network; MN, motor‐sensory network; SN, salience network; VN, visual network

Representative individual 18 network parcellation is shown in Figure 2a. Individual network sizes of the AN, DMN, FPN, LN, MN, SN, and VN were 539.87 ± 89.89, 631.70 ± 110.83, 987.56 ± 102.73, 446.23 ± 89.89, 1051.08 ± 96.28, 311.76 ± 77.18, and 535.35 ± 62.80 (mean ± SD), respectively. The change ratios of sizes were 0.14 ± 0.19 for AN, −0.13 ± 0.15 for DMN, 0.29 ± 0.13 for FPN, 0.39 ± 0.28 for LN, 0.11 ± 0.10 for MN, −0.08 ± 0.23 for SN, and 0.04 ± 0.12 for VN. The AIC showed that linear regression was more suitable than quadratic regression (absolute differences in AIC between linear and quadratic regression [AIC_diff_]: 1.55). As shown in Figure 2b, DMN (adjusted *r* ^2^ = .018, *p* < .001), FPN (adjusted *r* ^2^ = .025, *p* < .001), and SN (adjusted *r* ^2^ = .038, *p* < .001) showed significant negative correlations with age, while LN showed significant positive correlations with age (adjusted *r* ^2^ = .027, *p* < .001). No significant correlations were found in AN (adjusted *r* ^2^ = .004, *p* = .064), MN (adjusted *r* ^2^ = .004, *p* = .065), or VN (adjusted *r* ^2^ = .005, *p* = .064). In addition, GEE analysis showed significant differences in the change ratio of size between each pair of networks (all *p* < .01; Figure 2c).

### 3.2 Dynamic functional states

**FIGURE 3:**
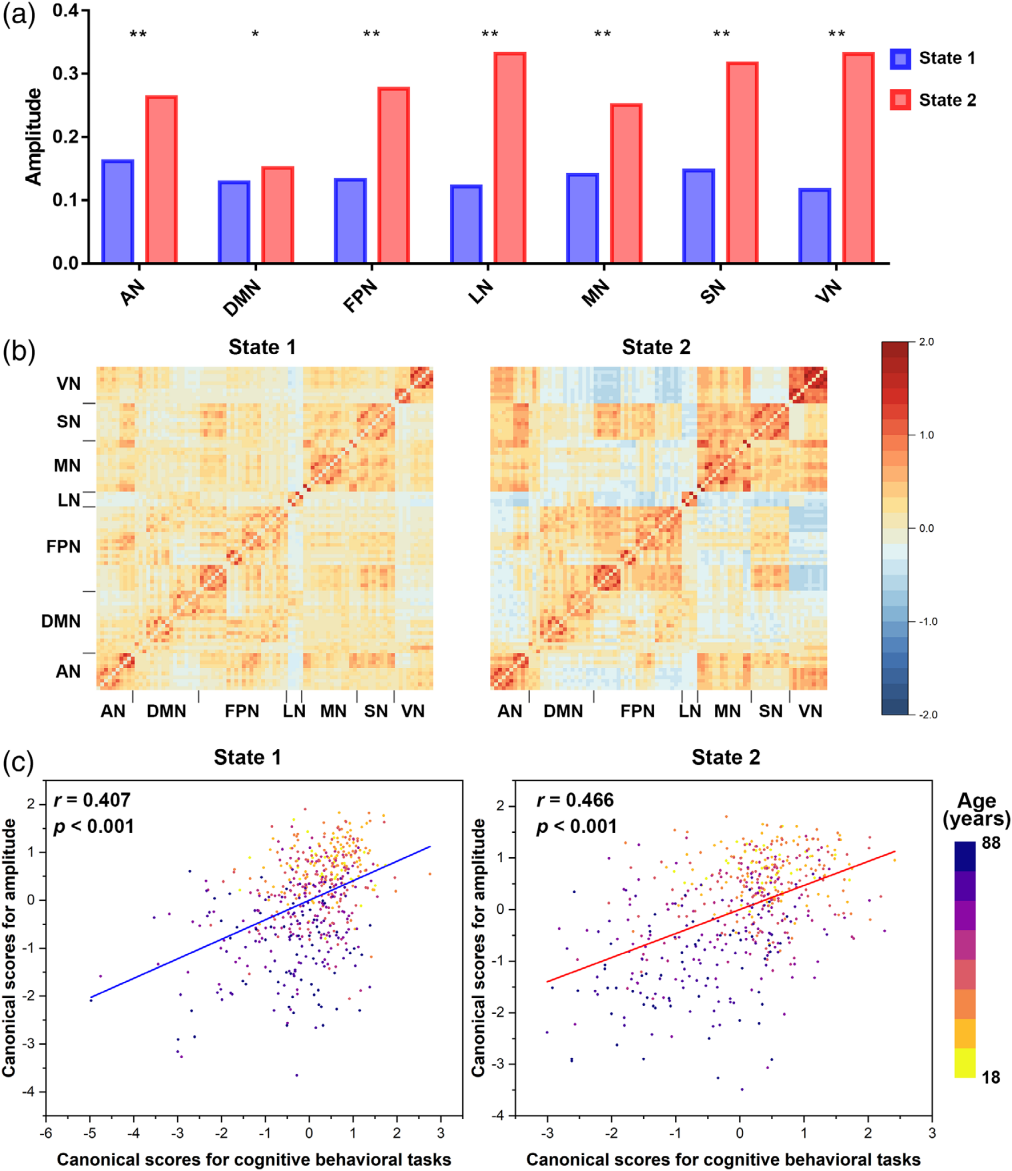
Differences in two cluster centres (states). (a) Median amplitude. Amplitude was compared by the Wilcoxon rank sum test. (b) Cluster centres (states). Different states were classified according to amplitude. (c) Canonical correlation analysis was performed between state amplitudes and cognitive behavioural tasks. Each participant was plotted as one dot. The colour of the dot indicates age. AN, attentional network; LN, limbic network; DMN, default mode network; FPN, frontoparietal network; MN, motor‐sensory network; SN, salience network; VN, visual network. * indicates *p* < .05; ** indicates *p* < .001

Medians of States 1 and 2 amplitudes were 0.16 and 0.26 for AN, 0.13 and 0.15 for DMN, 0.13 and 0.28 for FPN, 0.12 and 0.33 for LN, 0.14 and 0.25 for MN, 0.15 and 0.32 for SN, and 0.12 and 0.33 for VN (Figure 3a). The Wilcoxon rank sum test showed significantly high amplitudes for State 2 and low amplitudes for State 1 (all *p* < .05), indicating strong interactions for State 2. Clustering centroids are shown in Figure 3b as dynamic functional states. Since we performed clustering analysis for a given network‐related FC, the state matrix was asymmetric but approximately symmetric. As shown in Figure 3c, CCA showed strong positive correlations between amplitude and cognitive tasks in State 1 (*r* = .407, *p* < .001) and State 2 (*r* = .466, *p* < .001), and the correlation coefficients of State 2 were larger than those of State 1 (*z* = 1.83, *p* = .034), indicating that State 2 may support more cognitive tasks.

### 3.3 Dynamic functional state changes with ageing

**FIGURE 4:**
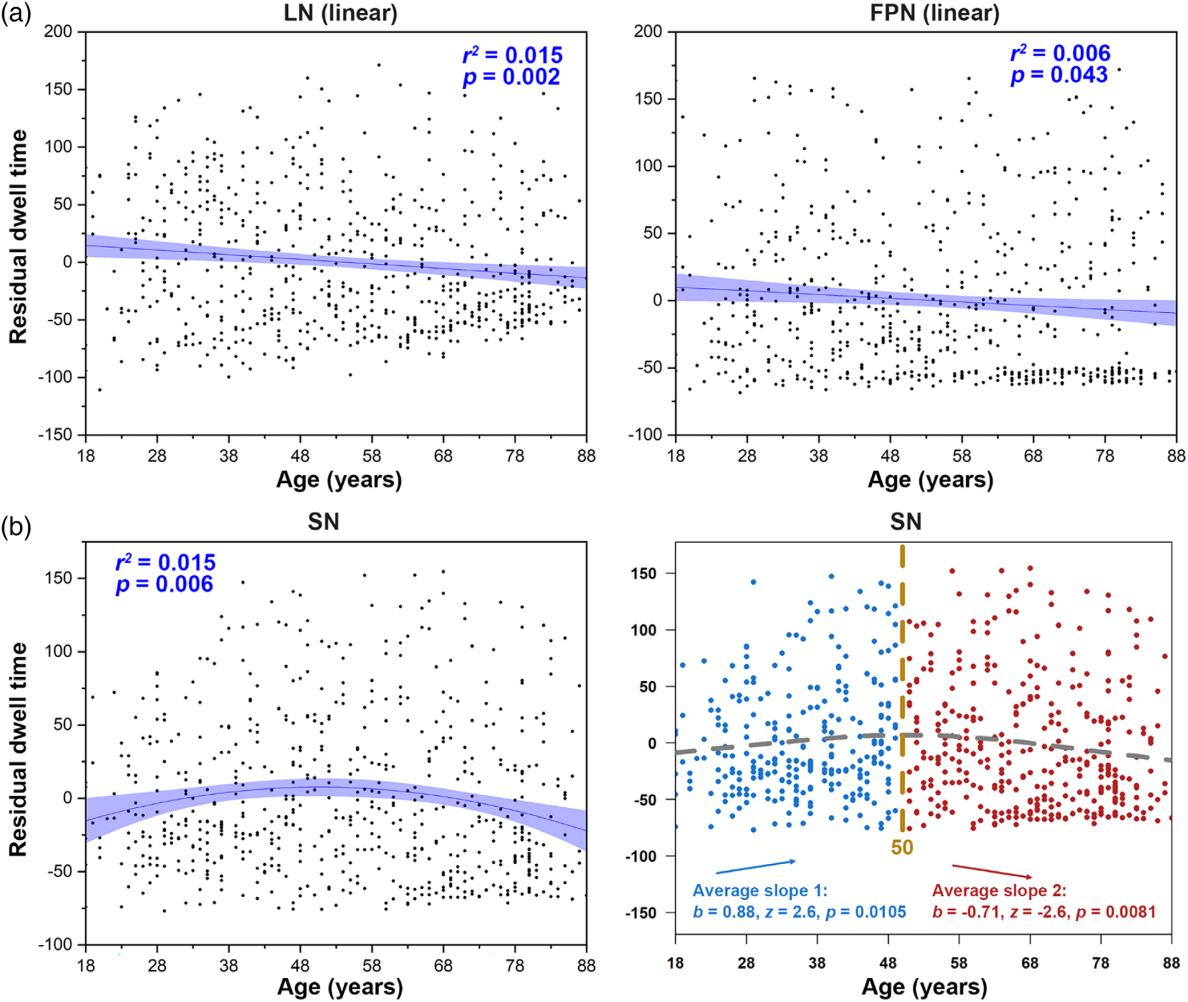
Ageing‐related changes in dwell time (State 1). (a) Linear decrease in dwell time (State 1) in the LN and FPN with increasing age. Linear regression *r* ^2^ and *p* values are shown, with sex, head motion, and cortical grey matter volume as covariates. (b) U‐shaped changes in the salience network (SN). Quadratic regression *r* ^2^ and *p* values are shown, with sex, head motion, and cortical grey matter volume as covariates. The “two‐lines” test showed an increase up to 50 years, followed by a decline. FPN, frontoparietal network; LN, limbic network; SN, salience network

To investigate changes in the two states with ageing, we examined how the temporal properties, including the mean dwell time, occurrence, and transition time, varied with age. For State 1 (low amplitude and cognitive correlations), the dwell time of LN (absolute differences in AIC between linear and quadratic regression, AIC_diff_ = 1.84) and FPN (AIC_diff_ = 1.23) showed significant negative linear correlations with age (Figure 4a). SN (AIC_diff_ = 8.15) showed significant quadratic correlations with age, and “two‐lines” tests were significant (Figure 4b). No ageing‐related correlations were found in dwell time of State 1 in AN (AIC_diff_ = 1.80; linear correlation with age: adjusted *r* ^2^ = −.002, *p* = .86), DMN (AIC_diff_ = 1.92; linear correlation with age: adjusted *r* ^2^ = −.0008, *p* = .67), MN (AIC_diff_ = 2.00; linear correlation with age: adjusted *r* ^2^ = −.001, *p* = .86) or VN (AIC_diff_ = 0.29; quadratic correlation with age: adjusted *r* ^2^ = .0005, *p* = .54).

**FIGURE 5:**
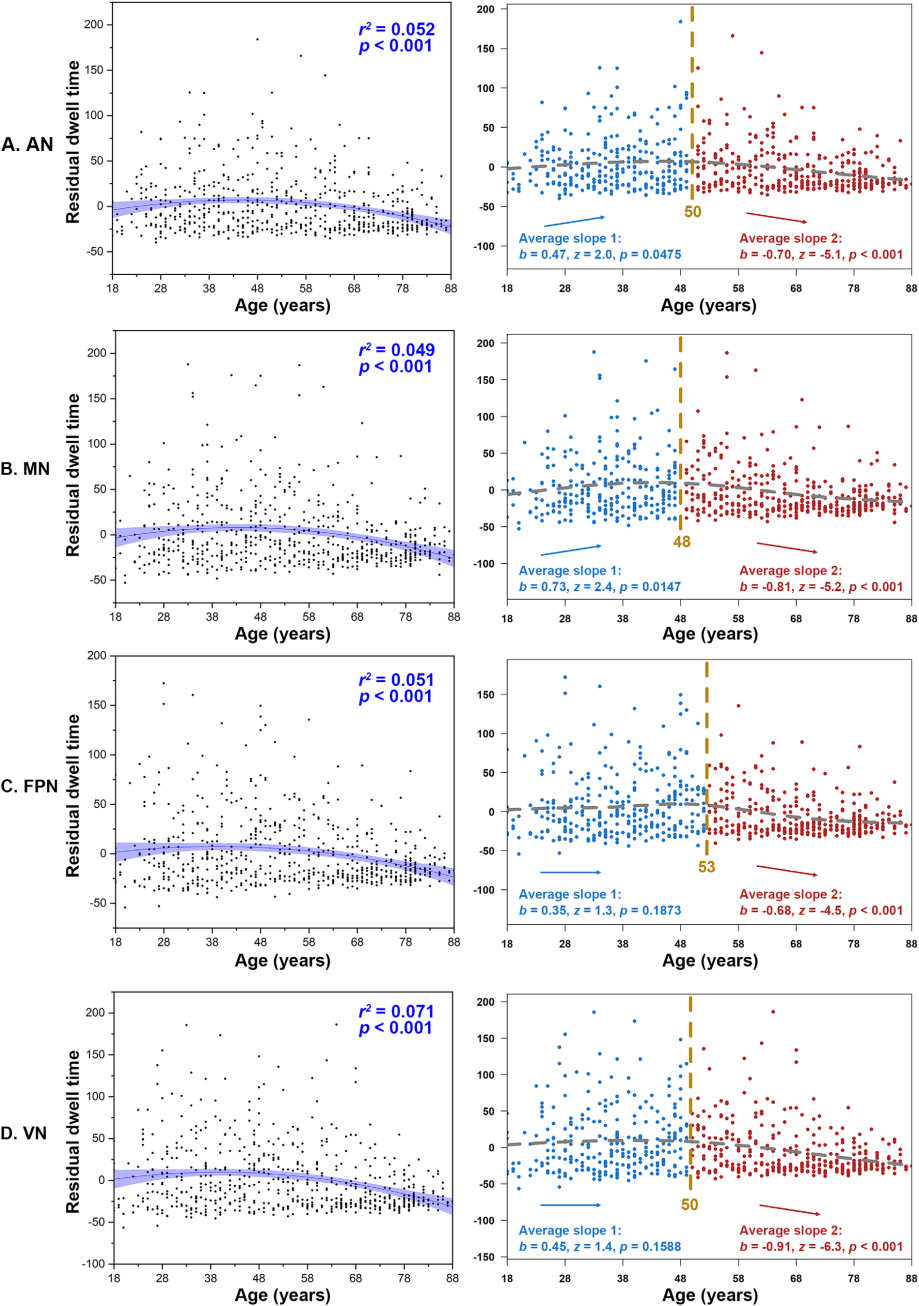
Ageing‐related U‐shaped changes in dwell time (State 2). (a–d) U‐shaped changes in AN, MN, FPN and VN, respectively. Quadratic regression *r* ^2^ and *p* values are shown, with sex, head motion, and cortical grey matter volume as covariates. (a) The “two‐lines” test showed an increase up to 50 years followed by a decline. (b) The “two‐lines” test showed an increase up to 48 years followed by a decline. (c) The “two‐lines” test showed a significant decrease after 53 years. (d) The “two‐lines” test showed a significant decrease after 50 years. AN, attentional network; FPN, frontoparietal network; MN, motor‐sensory network; VN, visual network

**FIGURE 6:**
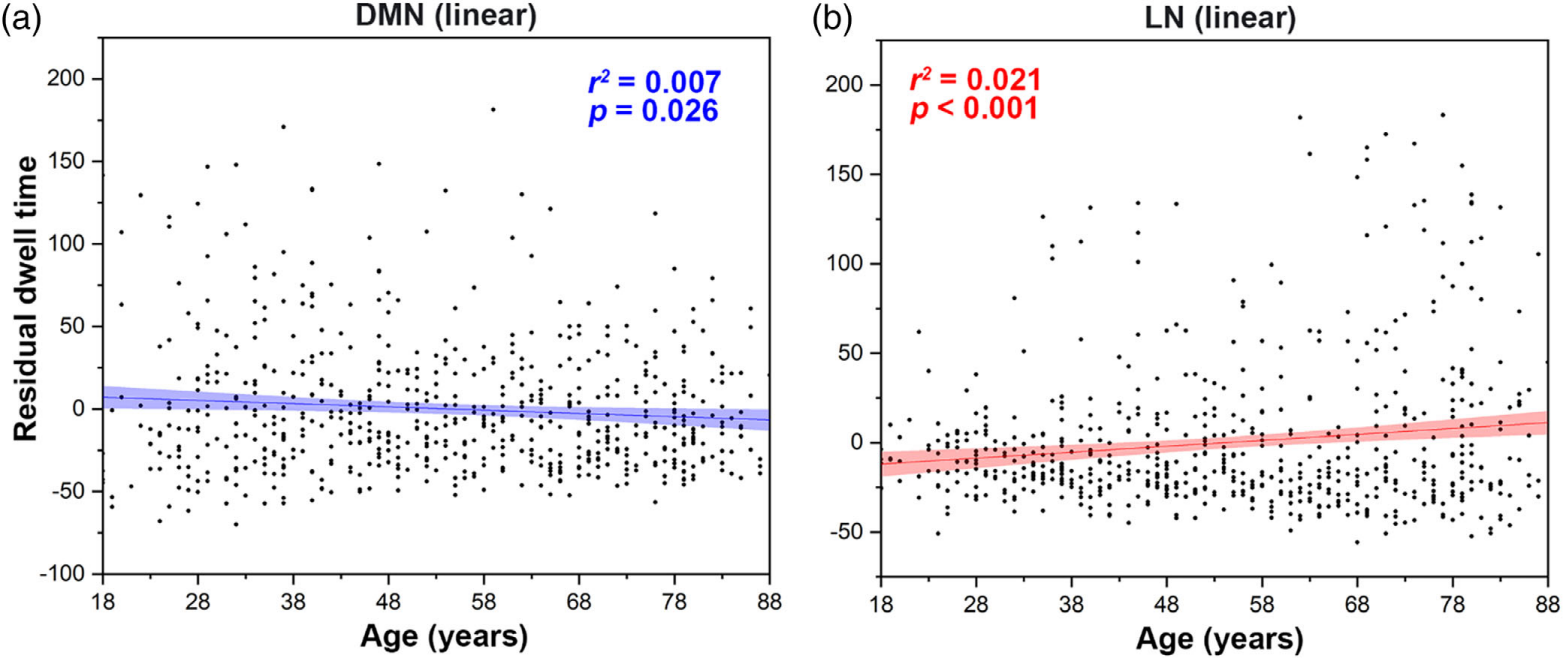
Ageing‐related changes in dwell time (State 2). (a) Linear decrease in dwell time (State 2) in the DMN with increasing age. (b) Linear increase in dwell time (State 2) in LN with increasing age. Linear regression *r* ^2^ and *p* values are shown, with sex, head motion, and cortical grey matter volume as covariates. AN, attentional network; DMN, default mode network; FPN, frontoparietal network; LN, limbic network; MN, motor‐sensory network; VN, visual network

For State 2 (high amplitude and cognitive correlations), dwell time of AN (AIC_diff_ = 14.52) and MN (AIC_diff_ = 12.02) showed significant quadratic correlations with age, and “two‐lines” tests were significant. FPN (AIC_diff_ = 7.13) and VN (AIC_diff_ = 11.40) also showed significant quadratic correlations with age, but “two‐lines” tests were not significant (Figure 5). The dwell time of the DMN (AIC_diff_ = 1.83) showed significant negative linear correlations with age, while the dwell time of the LN (AIC_diff_ = 0.25) showed significant positive linear correlations (Figure 6). No significant correlations were found in the dwell time of SN (AIC_diff_ = 1.18; linear correlation with age: adjusted *r* ^2^ = −.001, *p* = .68).

**FIGURE 7:**
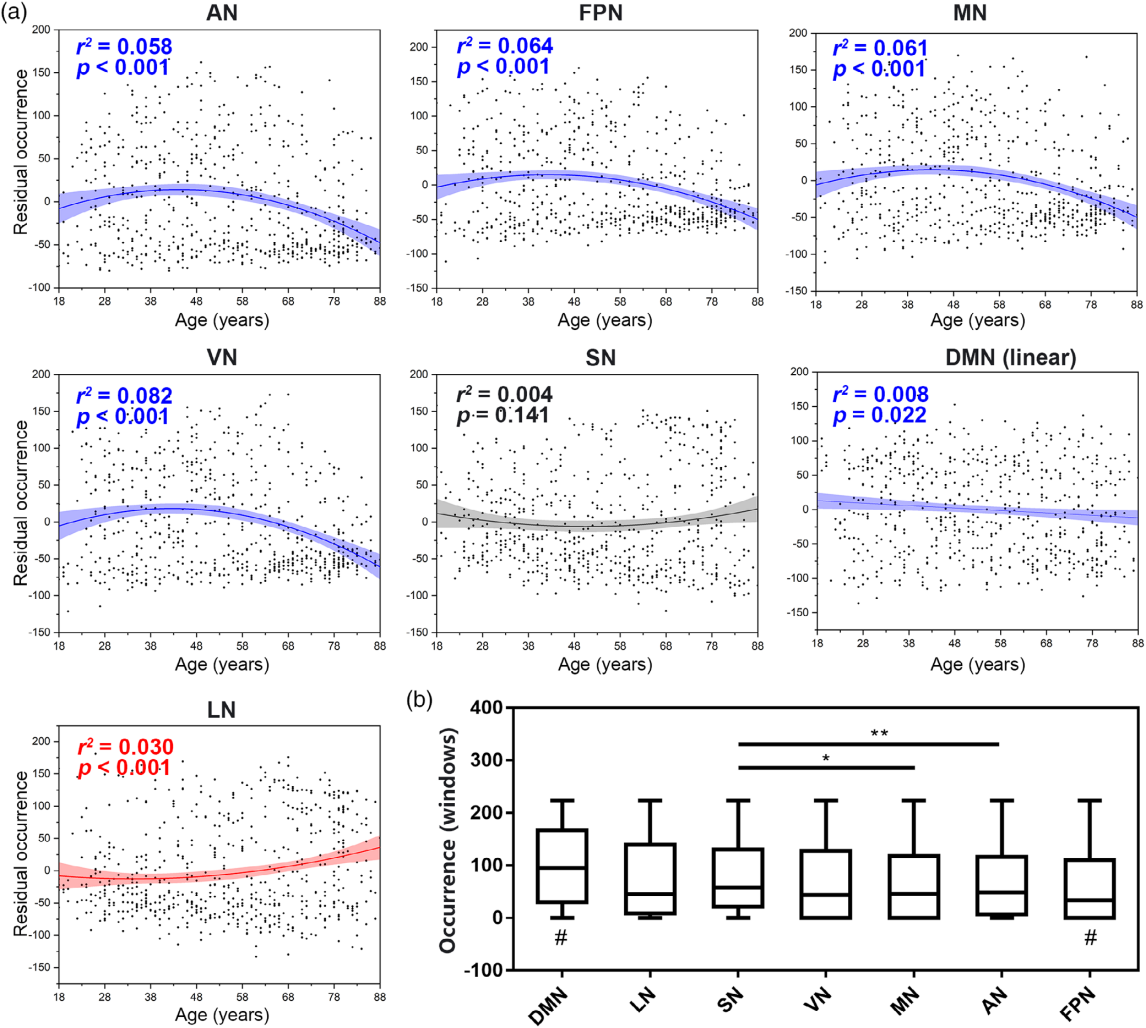
Ageing‐related changes of occurrence (State 2). (a) Changes in seven networks, including AN, FPN, MN, VN, SN, DMN, and LN. Quadratic regression *r* ^2^ and *p* values are shown, with sex, head motion, and cortical grey matter volume as covariates. (b) Comparisons of occurrence among different networks. Generalized estimated equation (GEE) analysis and post hoc comparisons. AN, attentional network; DMN, default mode network; FPN, frontoparietal network; LN, limbic network; MN, motor‐sensory network; SN, salience network; VN, visual network. * indicates *p* < .05; ** indicates *p* < .01. # indicates *p* < .05 when compared with any other network

Since the sum of occurrence of two states was constant (= 224 windows), their relationships with age were opposite, and the significance *p* value was the same. Thus, we only reported the results of the occurrence of State 2. The occurrence of State 2 was 65.45 ± 62.74 for AN, 98.90 ± 73.30 for DMN, 59.40 ± 65.59 for FPN, 76.05 ± 77.44 for LN, 65.85 ± 66.76 for MN, 80.13 ± 69.68 for SN, and 68.63 ± 70.20 for VN. The occurrence of the DMN showed a linear decrease with age (AIC_diff_ = 1.12; linear correlation with age: adjusted *r* ^2^ = .008, *p* = .022), and no significant correlations were found in the occurrence of SN (AIC_diff_ = 1.88; quadratic correlation with age: adjusted *r* ^2^ = .004, *p* = .141). Quadratic correlations were found in AN (AIC_diff_ = 16.13; quadratic correlation with age: adjusted *r* ^2^ = .058, *p* < .001), FPN (AIC_diff_ = 13.74; quadratic correlation with age: adjusted *r* ^2^ = .064, *p* < .001), MN (AIC_diff_ = 14.96; quadratic correlation with age: adjusted *r* ^2^ = .061, *p* < .001), VN (AIC_diff_ = 19.50; quadratic correlation with age: adjusted *r* ^2^ = .082, *p* < .001), and LN (AIC_diff_ = 1.50; quadratic correlation with age: adjusted *r* ^2^ = .030, *p* < .001). Of these, only the correlations of LN first decreased and then increased (Figure 7). GEE showed that the occurrence of the DMN was higher than that of any of the other six networks (all *p* < .001), and the occurrence of the FPN was lower than that of any of the other six networks (all *p* < .05). In addition, the occurrence of SN was larger than that of MN (*p* = .015) and AN (*p* = .009).

**FIGURE 8:**
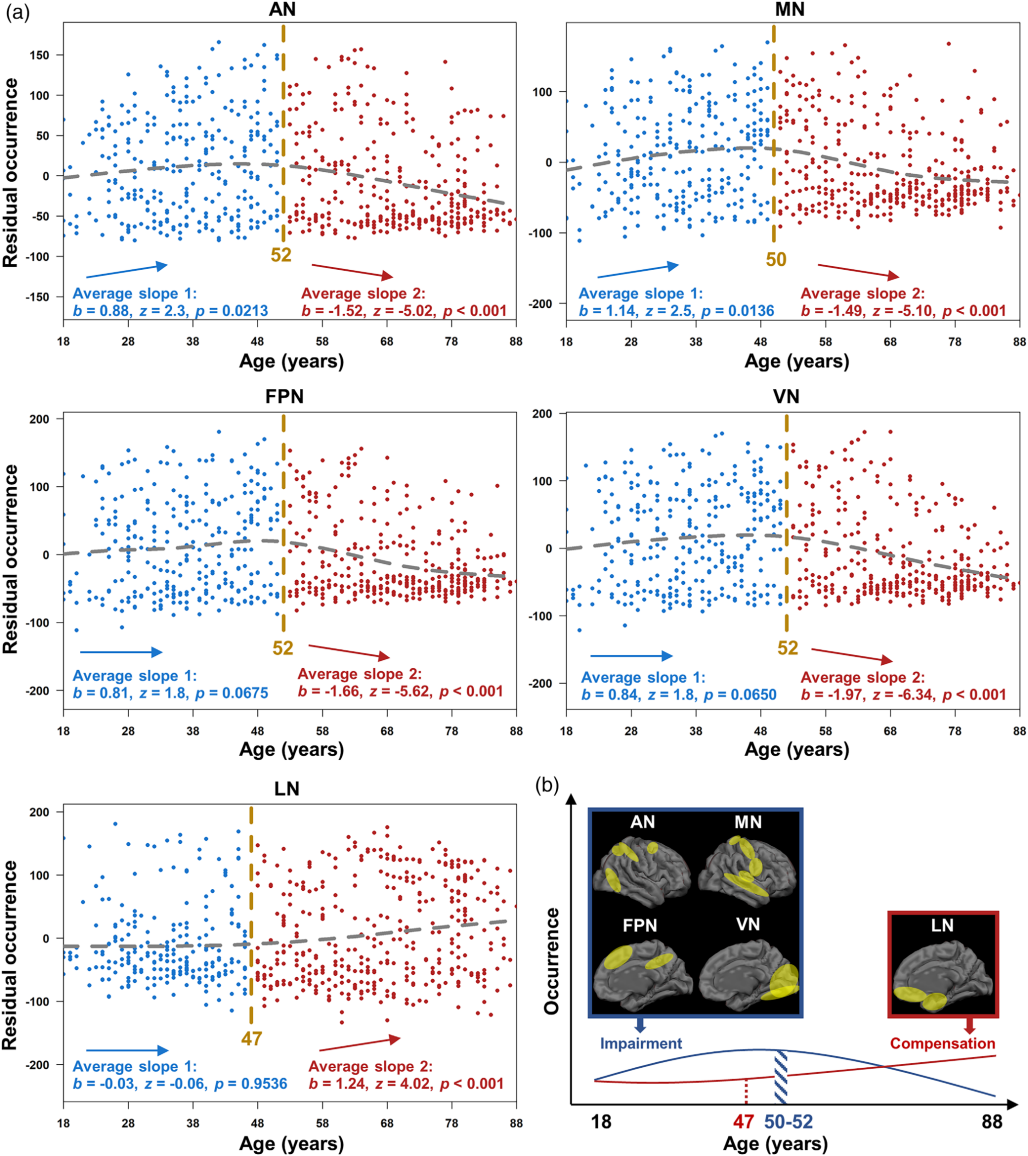
U‐shaped changes of occurrence (State 2). (a) The “two‐lines” test for AN, MN, FPN, VN, and LN. The “two‐lines” test showed significant upward and downward slopes in AN and MN. No significant upward slope was found in the FPN, VN, or LN. (b) Scheme of the impairment and compensation process. The blue line indicates the impairment process of AN, MN, FPN, and VN. The red line indicates the compensation process of LN. AN, attentional network; FPN, frontoparietal network; LN, limbic network; MN, motor‐sensory network; VN, visual network

Significant inverted U‐shaped patterns were found in AN (upward slope: *p* = .0213; downward slope: *p* < .001) and MN (upward slope: *p* = .0136; downward slope: *p* < .001). The occurrences of FPN (upward slope: *p* = .0675; downward slope: *p* < .001) and VN (upward slope: *p* = .0650; downward slope: *p* < .001) were marginally significant, but LN was not significant (upward slope: *p* = .9536; downward slope: *p* < .001). Finally, in terms of the occurrence of State 2, we proposed a model to describe the impairment and compensation of AN, MN, FPN, VN, and LN with ageing (Figure 8). Inverted U‐shaped correlations with age were defined as impairment processes, and U‐shaped correlations with age were defined as compensation processes.

## 4 DISCUSSION

The present study used individual ROI parcellation and individual‐length sliding window analysis to characterize dynamic variations in individual functional network states across the lifespan (18–88 years). The individual parcellation captured individual variations and revealed a linear decrease in the DMN, SN, and FPN and a linear increase in LN with increasing age, indicating atrophy of most cognitive functional networks but expansion of emotion‐related LN in healthy ageing. In addition, sliding windows and *k*‐means clustering analysis were applied to each of seven networks, including AN, MN, FPN, VN, DMN, LN, and SN, and revealed two recurring brain states for each network. One was strong, characterized by strong FC amplitude and strong correlations with cognitive tasks, and the other was weak, characterized by weaker FC amplitude and weaker correlations with cognitive tasks than the strong state. In terms of weak state analysis, LN and FPN dwell time showed a linear decrease, and SN dwell time showed an inverted U‐shaped correlation with age, suggesting the difficulties of maintaining the same brain states in the healthy ageing process. In terms of strong state analysis, DMN occurred and dwelled less with increasing age; FPN and VN began to occur and dwell less in middle age (50–53 years); AN and MN occurred and dwelled more before middle age (48–52 years) followed by a decline. These findings suggest that in the healthy ageing process, the resting brain may allocate insufficient time to the strong state, characterized by a high correlation with cognitive function, which may further lead to inefficient cognitive resource allocation. In addition, we have shown that the LN strong state dwells more with increasing age and occurs more in middle‐aged adults (older than 47 years), indicating the compensation of the LN strong state for the impaired process of most functional network states.

In this study, individual cortical parcellation was mapped using established group‐level parcellation (Yeo et al., 2011) and each participant's BOLD signal variations. Functional network size indicates the number of vertices assigned to the same network in individual parcellation relative to the number of vertices in group parcellation. Similar to prior studies using group‐level network parcellations (Bajaj et al., 2017; Lowe et al., 2019; Westlye et al., 2010), our study provides support for brain atrophy with age. Based on our observations, the change ratios of sizes in high‐order association networks, including the DMN, SN, and FPN, decreased with increasing age, indicating a shrunken cortex across the adult lifespan. In comparison, the change ratio of sizes in primary sensory networks, including the MN and VN, is relatively maintained. These findings support the theory that late‐developing networks (i.e., high‐order networks) are sensitive to the ageing process (Douaud et al., 2014; T. Liu et al., 2022; Westlye et al., 2010). Notably, we found a significant positive linear association between age and the change ratio of LN size, suggesting that LN size may consistently expand across the adult lifespan. It has been suggested that changes in the LN are different from other high‐order cognitive networks during development and the ageing process. One previous early adult (18–45 years) study showed that the cortical thickness of primary sensory and high‐order networks is negatively correlated with age, except for the LN (Bajaj et al., 2017). In addition, one previous study mapped group parcellation of children and compared it to adult parcellation (Yeo's parcellation (Yeo et al., 2011)). They found that the parcellation of primary sensory networks is similar, but the DMN regions in adult parcellations are partially assigned to the LN in children (Tooley et al., 2022). These findings support our results that three main cognitive networks, including the DMN, FPN, and SN, shrink, but the emotional network, that is, the LN, expands across the adult lifespan.

At the temporal scale, this study revealed one strong state, characterized by strong FC amplitude and strong correlations with cognitive functions, and one weak state, characterized by weak FC amplitude and weak correlations with cognitive functions. Temporal metrics were calculated for each state, including occurrence, indicating how often a state occurs, and dwell time, indicating how much time is spent in a state before transitioning to the other state. In our study, the two states were clustered by within and between FC of each network, resulting in 14 states (7 networks × 2 states). Weak states of three networks (LN, FPN, and SN) showed significant correlations with age, and strong states of six networks (AN, MN, FPN, VN, DMN, and LN) showed significant correlations with age. These findings suggest that strong brain states, characterized by strong FC amplitude and strong correlations with cognitive functions, are more vulnerable than weak brain states. Correlations between dynamic brain states and cognitive functions have been reported in a previous study (Vidaurre et al., 2017). Healthy ageing studies have further revealed the weak state and showed increased dwell time of the weak state with increasing age (K. Y. Chen et al., 2022; Tian et al., 2018), supporting the stability of the weak state and the vulnerability of the strong state.

In this study, the occurrence and dwell time of AN and MN strong states and the dwell time of SN weak states showed inverted U‐shaped correlations with age. Previous studies have also shown nonlinear correlations between temporal parameters and age (K. Y. Chen et al., 2022; Kupis et al., 2021; Snyder et al., 2021), but these studies do not further test the validity of U‐shaped correlations, that is, whether the upward and downward slope is significant. In this study, we performed a post hoc “two‐lines” test and revealed an inverted U‐shaped model with maximum temporal parameters occurring at approximately 50 years (i.e., an upward slope before approximately 50 years followed by a downward slope). We also found that the occurrence and dwell time of the VN strong state is nonlinearly correlated with age, but the upward trend is not significant, suggesting that the VN strong state occupies less time with increasing age after approximately 50 years. In addition, the peak age is supported by a previous study (participants: 21–86 years), which reported that structural intrinsic brain volume changes exhibit an inverted U‐shaped correlation with peaks occurring at approximately 45 years of age (Bagarinao et al., 2018).

The FPN, DMN, and SN are three key networks related to ageing (Damoiseaux et al., 2008; Hardcastle et al., 2022; Oschmann, Gawryluk, & Alzheimer's Disease Neuroimaging Initiative, 2020). This study revealed SN changes in weak states and DMN changes in strong states, indicating the state dependence of ageing‐related changes in the SN and DMN. Different dynamic states of the SN have been examined and showed quadratic relationships with age (Snyder et al., 2021). In addition, DMN‐dominant states have shown linear relationships with age (T. Liu et al., 2022) and reduced occurrence in participants with subjective cognitive decline (Liang et al., 2021). Different from the state dependence of SN and DMN, the FPN exhibits decreasing dwell time in terms of both strong and weak states, indicating the wide functional changes of the FPN. The FPN is a flexible hub in the brain, and one previous study showed a quadric relationship between FPN state and age (Kupis et al., 2021). In addition, FPN changes can be modulated by cognitive training in older adults (65–84 years) (Hardcastle et al., 2022).

With the decreased occurrence and dwell time of most networks, our results showed a compensative increase in the LN in terms of strong state analysis. The LN includes the bilateral anterior temporal lobe, medial temporal lobe, subgenual anterior cingulate cortex, and medial and lateral orbitofrontal cortex (Oosterwijk et al., 2012) and plays a significant role in emotional and reward‐related processing (Cao et al., 2021; Rolls, 2019). Compared to other higher‐order and primary sensory networks, the LN has shown the largest functional network pattern changes during the first 6 years of life (H. T. Chen et al., 2021). An independent component analysis compared FC differences between young (18–33 years) and old (58–85 years) adults and found differences in cognitive networks but not in emotion networks (Nashiro et al., 2017), suggesting cognitive function decline and emotional function maintenance with age. In line with these results, we found that the LN strong state occurred and dwelled more with increasing age, indicating the compensative function of the LN. This is supported by a local FC analysis on the same dataset, which also found decreased local FC within the VN, SMN, and DMN but increased local FC within the basal ganglia network with increasing age (Wen et al., 2020). In addition, one study analysed baseline, 1‐, 2‐, 3‐, and 4‐year follow‐up rs‐fMRI data from old adults (64–87 years) and reported decreased segregation of the DMN, FPN, and SN and an increase in the LN (Malagurski et al., 2020). Overall, compared to the decline in other cognitive networks, such as the DMN, FPN, and SN, the LN may maintain and even compensatively increase with increasing age (Nashiro et al., 2012; Scheibe & Carstensen, 2010).

Several limitations should be considered when interpreting these findings. First, this study reports ageing‐related changes in a group of participants, not within individuals, as individual studies require longitudinal follow‐up. Second, this rs‐fMRI dataset collected 261 volumes (8 min and 40 s) for each participant, and more volumes would result in more precise individual parcellation (D. H. Wang et al., 2015). However, the relatively less precise parcellation is tolerable compared to group parcellation in ageing studies (Geerligs et al., 2017). Third, we clustered all dynamic FCs into two brain states, as done in previous studies (Kim et al., 2017). However, some studies clustered more brain states, such as three (Tian et al., 2018) and five (Xia et al., 2019). Perhaps each participant has an individual number of recurrent brain states, but this would make group comparisons more difficult. Thus, more research is needed to describe individual brain states.

## 5 CONCLUSION

In summary, this study used individual functional parcellation and individual sliding window clustering to reveal brain functional changes across the lifespan. Individual parcellations showed that the sizes of the DMN, FPN and SN decreased with increasing age, accompanied by an increase in LN size. In terms of individual sliding window clustering analysis, we revealed two brain states for each functional network, in which one has strong FC amplitudes and strong correlations with cognitive functions, called the strong state, and the other is the weak state. Temporal clustering showed that ageing‐related decline was common in strong states and late adulthood (older than 47–53 years). Notably, the FPN showed a decreasing pattern in both strong and weak states, and only LN strong states showed an increasing pattern across the lifespan. Overall, our findings highlight the compensative function of the LN in functional network size and dynamics with age.

## AUTHOR CONTRIBUTIONS

Tiantian Liu and Zhongyan Shi: Conceptualization, formal analysis, methodology, validation, visualization, writing ‐ original draft, review and editing. Jian Zhang: Methodology, writing ‐ review and editing. Kexin Wang: Methodology, writing ‐ review and editing. Yuanhao Li: Writing ‐ review and editing. Guangying Pei: Writing ‐ review and editing. Li Wang: Writing ‐ review and editing. Jinglong Wu: Writing ‐ review and editing. Tianyi Yan: Supervision, resources, writing ‐ review and editing.

## FUNDING INFORMATION

This work was supported by the National Natural Science Foundation of China (grant numbers 61727807, U20A20191, 82071912, 12104049); the Beijing Municipal Science and Technology Commission (grant number Z201100007720009); the Fundamental Research Funds for the Central Universities (grant number 2021CX11011); the China Postdoctoral Science Foundation (grant number 2020TQ0040); and the National Key Research and Development Program of China (grant number 2020YFC2007305). The Cambridge Centre for Ageing and Neuroscience (Cam‐CAN) provided study data. Cam‐CAN funding was provided by the UK Biotechnology and Biological Sciences Research Council (grant number BB/H008217/1), together with support from the UK Medical Research Council and University of Cambridge, UK.

## CONFLICT OF INTEREST

The authors declare no conflicts of interest.

## PATIENT CONSENT STATEMENT

All participants gave written informed consent.

## Data Availability

Data were provided by the Cambridge Centre for Ageing and Neuroscience (Cam‐CAN) project (http://www.mrc-cbu.cam.ac.uk/datasets/camcan/). Codes are available in the GitHub repository (https://github.com/Tiantian-LIU-BIT/Individual_Net_clustering.git).
